# Sex-related peripheral immune profile in ulcerative colitis: links to fatigue

**DOI:** 10.3389/fimmu.2026.1824822

**Published:** 2026-05-14

**Authors:** Thyra Albrecht, Elisa Wider-Eberspächer, Alejandra P. Garza, Lorena Morton, Sabrina Sulzer, Verena Keitel-Anselmino, Rosa Rosania, Ildiko R. Dunay

**Affiliations:** 1 Institute of Inflammation and Neurodegeneration, Medical Faculty, Otto-von-Guericke University Magdeburg, Magdeburg, Germany; 2 Department of Gastroenterology, Gastrointestinal Oncology and Endocrinology, University Medical Center, Goettingen, Germany; 3 Department of Gastroenterology, Hepatology and Infectious Diseases, University Hospital Magdeburg, Magdeburg, Germany; 4 Center for Behavioral Brain Sciences, Magdeburg, Germany; 5 German Center for Mental Health (DZPG), Magdeburg, Germany; 6 Centre for Intervention and Research on Adaptive and Maladaptive Brain Circuits Underlying Mental Health (C-I-R-C), Magdeburg, Germany

**Keywords:** adaptive immunity, dendritic cells, fatigue, inflammatory cytokines, innate immunity, monocytes, neuroinflammation, sex differences

## Abstract

**Background:**

Ulcerative colitis (UC) is a chronic inflammatory disease marked by mucosal and systemic immune dysregulation. Fatigue is a common, burdensome extraintestinal symptom that often persists beyond active inflammation and affects quality of life. Sex-based differences in immune response and fatigue severity have been reported, but their mechanistic basis remain unclear. This study aimed to characterize peripheral immune profiles across UC disease stages and explore links between immunity, fatigue, and sex.

**Methods:**

Eighty-nine individuals were enrolled: active UC (A; n=29), remission (R; n=30), and healthy controls (C; n=30). Flow cytometry assessed peripheral neutrophils, monocytes, dendritic cells (DCs), and T cell subsets, alongside plasma cytokines (BDNF, TNF, IL-6, IL-18, sTREM-2). Fatigue was evaluated using the validated Inflammatory Bowel Disease Fatigue (IBD-F) questionnaire, with sex-stratified correlation analyses.

**Results:**

Active UC was associated with increased neutrophils and classical monocyte, alongside elevated TNF, BDNF, and sTREM-2. Upregulated CD62L expression and CCR2 expression in neutrophils and monocyte subsets indicated ongoing immune cell trafficking. During remission, increased plasmacytoid and CD141^+^ DCs, and Th9/Th22 cells suggested protective immune modulation. Fatigue severity correlated with specific immune subsets in a sex-dependent manner: in males, fatigue inversely correlated with classical monocytes and Th9/Th17/Tregs; in females, with NK cells and Tregs. Fatigue scores were higher in female patients.

**Discussion:**

Peripheral immune dysregulation in UC correlates with fatigue severity in a sex-specific manner. Our findings underscore the relevance of gut-brain axis signaling and highlight immune biomarkers with potential for stratified fatigue management in UC.

## Highlights

- Ulcerative colitis is associated with distinct peripheral immune shifts across disease stages, involving both innate and adaptive immune compartments.
- Fatigue severity correlates with specific immune cell subsets in a sex-dependent manner in UC patients.
- Male UC patients display a proinflammatory Th17/Th2 signature, while females show persistent neutrophilia during remission.
- Fatigue may reflect unresolved systemic immune dysregulation and warrants consideration as a clinical monitoring tool in UC.

## Introduction

Ulcerative colitis (UC) is a chronic, relapsing inflammatory bowel disease (IBD) marked by mucosal and immune dysregulation (1). It impacts both innate and adaptive immune compartments, with altered immune cell populations in peripheral blood and intestinal mucosa (2).

Fatigue is a prevalent extraintestinal symptom in UC, affecting up to 68% of patients during active disease and 40% during remission (3). It represents persistent, multidimensional exhaustion not relieved by rest, and includes physical, cognitive, and affective components (4, 5). While anemia, nutrient deficiencies, medications and psychological comorbidities (such as anxiety and depression) contribute to fatigue, elevated inflammatory markers such as TNF, IFN-y and IL-10 have also been linked to fatigue in UC (6).

The gut-brain-axis, encompassing neural, immune and endocrine pathways, mediates bidirectional communication between the gastrointestinal tract and the brain. In UC, gut dysbiosis and chronic inflammation may impair this communication, contributing to fatigue, mood alterations and cognitive symptoms (7, 8).

Sex differences also modulate immune responses in health and disease (9). In UC, males and females differ in disease course and symptom burden: women more often report fatigue and mood disorders, while men may face higher surgery rates and earlier mortality (10). Hormonal fluctuations and sex-based pharmacokinetics may further impact disease activity and treatment response (4, 11). However, the immunological underpinnings of these sex-specific outcomes remain poorly defined.

This study aims to characterize peripheral immune cell profiles across UC disease stages and investigate their relationship with fatigue severity. We further explore whether sex modulates these immune-fatigue interactions, with attention to neuroimmune markers involved in gut-brain axis signaling. By integrating flow cytometry, cytokine analysis, and validated fatigue assessment, we seek to identify immune signatures associated with symptom burden and sex-specific disease mechanisms.

## Materials and methods

Extended experimental protocols, antibody panels, gating strategies, and full inclusion/exclusion criteria are provided in **Supplementary Methods (Supplementary Data Sheet S1)**.

### Study design and participants

**Figure 1:**
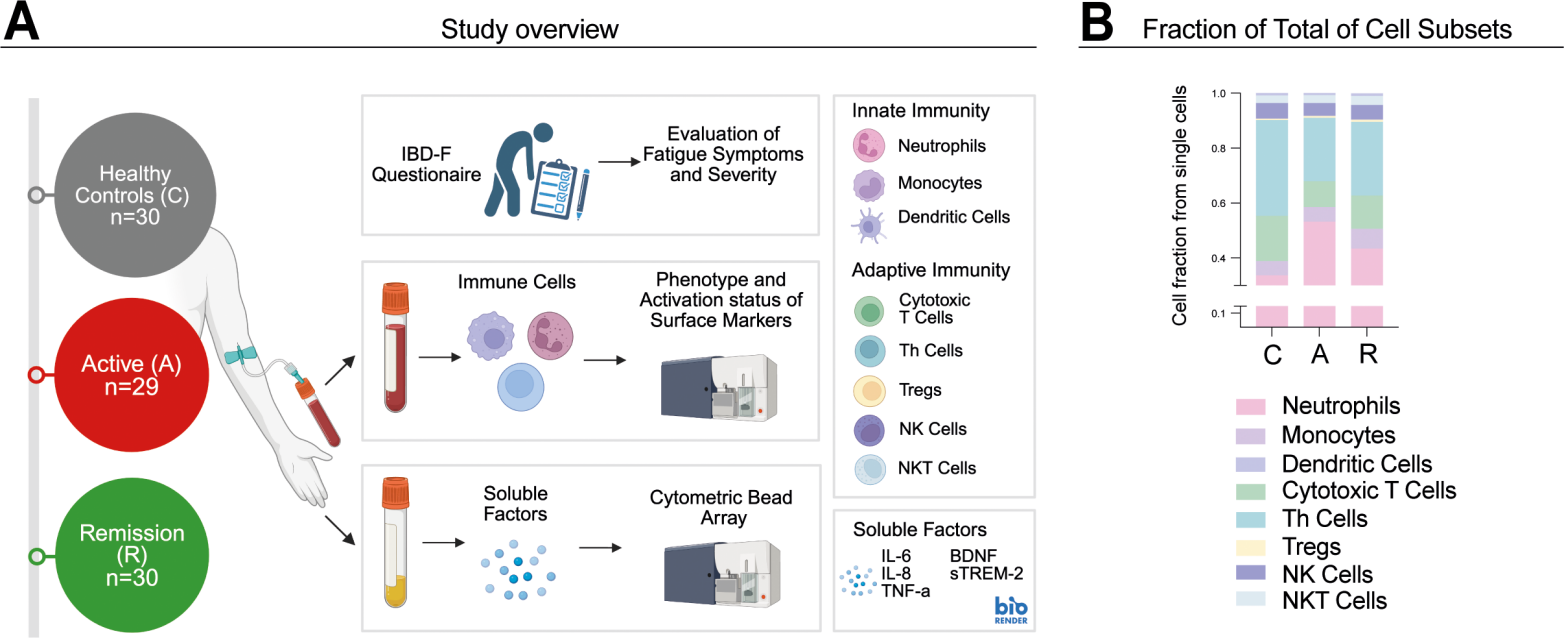
Experimental design and cell fractions. Detailed description of experimental design. Blood was sampled from UC patients in either the active state (n=30) (A) remission (n=29) (R) or from healthy controls (n=30) (C). Blood samples were prepared for the isolation of innate and adaptive immune cells, as well as for the assay of soluble factors. Additionally, participants completed fatigue questionnaires **(A)**. Panel **(B)** presents a bar chart showing the fractions of total cell subsets isolated from peripheral blood.

**Figure 2:**
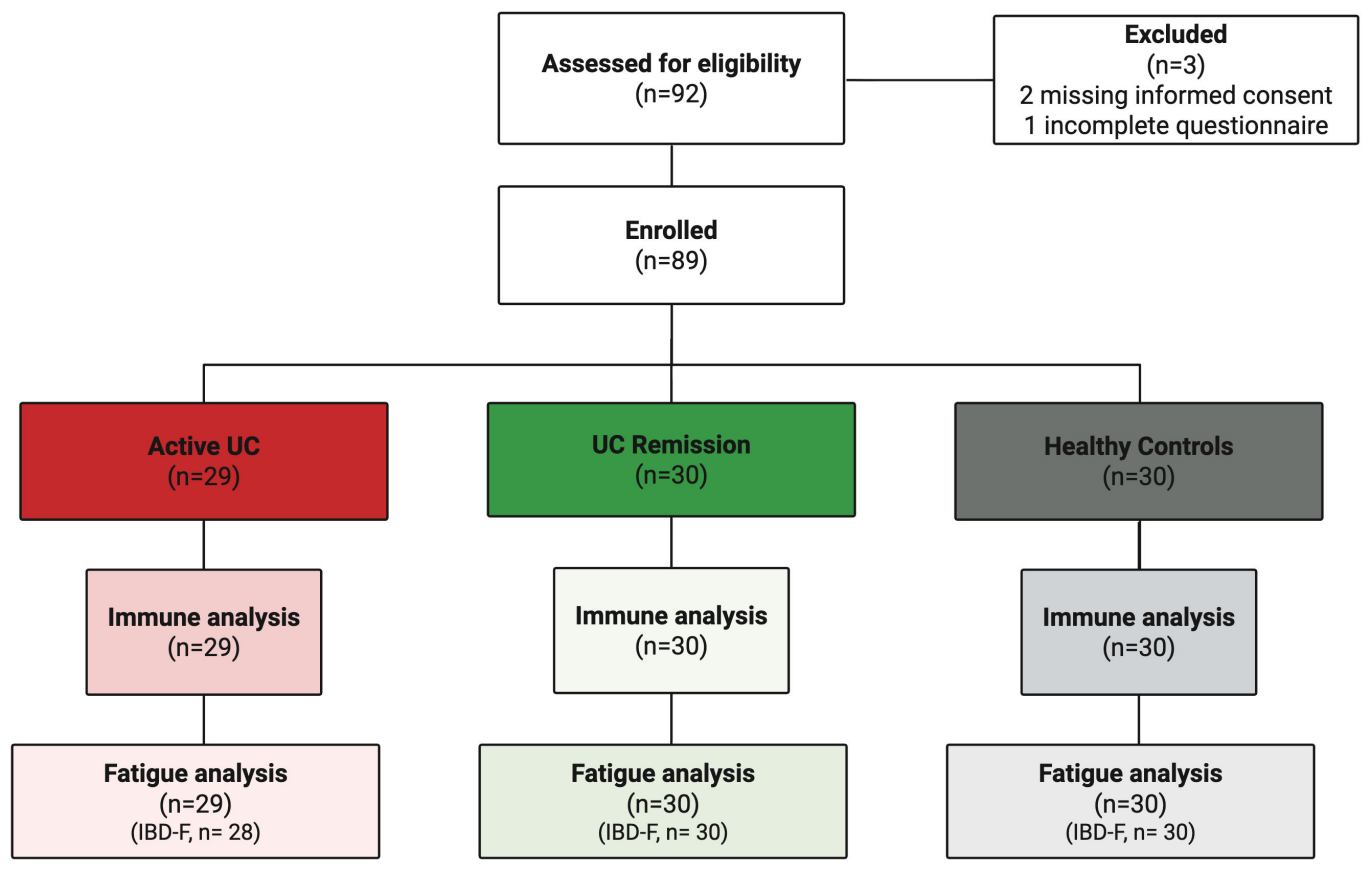
Participant flow diagram. Flow diagram illustrating participant recruitment, enrollment, and allocation to study groups. Immune profiling was performed for all enrolled participants. Fatigue assessment using the Inflammatory Bowel Disease Fatigue questionnaire (IBD-F) was completed by 88 of 89 participants; one participant in the active UC group did not return the fatigue questionnaire.

We enrolled 89 adults with UC or age- and sex-matched healthy controls (HC) from the University Clinic Magdeburg (Jan–Oct 2021), categorized into active UC (n=29), remission (n=30), or HC (n=30). A total of 92 individuals were assessed for eligibility; three were excluded prior to enrollment (two missing informed consent, one incomplete questionnaire), resulting in a final cohort of 89 participants. Study overview is shown in **Figure 1A**, while participant flow is summarized in **Figure 2**. A demographic overview is provided in **Table 1**. Active UC was defined as a partial Mayo score >2 and fecal calprotectin >250 µg/g; remission was defined as a partial Mayo score ≤2 and calprotectin <250 µg/g, a threshold reflecting real-world clinical practice that may not fully exclude residual mucosal inflammation. Exclusion criteria included major neurological, psychiatric, or inflammatory comorbidities.

**Table 1:** Demographic information.

Parameter	UC		Healthy controls	*p* value
	Active	Remission		
n	29	30	30	–
Sex (m/f)	15 (51%)/14 (48%)	14 (46%)/16 (53%)	12 (40%)/18 (60%)	0.5356
Age (M ± SD)	44.48 ± 11.53	41.50 ± 11.11	38.43 ± 11.63	0.1103^a^ 0.5540^b^ 0.5772^c^
Age of Onset years (M- Range)	33.28 (17-58)	29.15 (14-57)	–	0.1921^c^
Disease Duration (months) (M ± SD)	10.72 ± 9.11	11.52 ± 8.72	–	0.7481^c^
Smoking Status (Never/Ex-Smokers/Smokers)	9 (31%)/17 (58%)/3 (10%)	14 (46%)/11 (36%)/5 (16%)	27 (90%)/3 (10%)/0 (0%)	–
BMI (M ± SD)	24.65 ± 3.21	25.38 ± 3.62	24.65 ± 3.21	0.8551^a^ 0.7110^b^ 0.9629^c^
Partial Mayo-Score (M ± SD)	6.31 ± 2.06	1.33 ± 1.18	–	**<0.0001**
Montreal Classification (E1/E2/E3)	5 (17%)/17 (58%)/7 (24%)	4 (13%)/10 (33%)/15 (50%)	–	–
Drug history (untreated/local^1^ therapy/systemic^2^ therapy/unknown)	3 (10%)/11 (27%)/10 (34%)/5 (17%)	4 (13%)/15 (50%)/8 (26%)/3 (10%)	–	–

^1^only mesalazine; ^2^glucocorticoids, azathioprine, Jak inhibitors and biologicals (infliximab, vedolizumab, ustekinumab); ^a^control vs active, ^b^control vs remission, ^c^active vs remission.

An overview of the demographic data for the three groups: active (A), remission (R), and healthy controls (C), including categorization based on the Mayo Score and Montreal Classification.Bold values indicate statistical significance.

### Flow cytometry and immune profiling

Whole blood was processed via red blood cell lysis and stained with a 30-marker antibody panel targeting myeloid, lymphoid, and dendritic cell populations. Flow cytometry was performed on an Attune NxT instrument and analyzed using FlowJo v10.3. Cell gating strategies identified neutrophils, monocyte subsets, DCs, and CD4/CD8 T cell populations including Th subsets and Tregs.

### Cytokine analysis

Plasma was isolated via Ficoll density gradient and stored at −80 °C. Cytokines (TNF, IL-6, IL-18), BDNF, and sTREM-2 were measured using LEGENDplex bead-based multiplex assays and quantified using standard curves. For values below detection, half of the LOD was used.

### Fatigue assessment

All participants completed the IBD-F questionnaire (German version), assessing fatigue severity (Part I) and impact (Part II). Fatigue was stratified as none, moderate, or severe based on validated scoring structure **(Supplementary Data Sheet S1)**.

### Statistical analysis

Comparisons were made between the three study groups using ANOVA or Kruskal-Wallis tests, depending on data distribution. Data normality was assessed using the Shapiro-Wilk test prior to group comparisons. Sex, age, and BMI were assessed as potential confounders; as no significant differences were found between groups, they were not included as covariates. No a priori power calculation was performed; sample size was determined by recruitment availability. Sex-stratified Spearman rank correlations were used to assess associations between immune cell populations, plasma markers, and fatigue; these analyses were considered exploratory and results should be interpreted as hypothesis-generating and require validation in larger cohorts. Significance was set at p<0.05. All analyses were performed in GraphPad Prism 9.0.

## Results

### Peripheral immune alterations in UC

The innate immune system is central to UC pathogenesis, with neutrophils, monocytes, and dendritic cells (DCs) contributing to both inflammation and resolution (12). We first examined systemic immune alterations across disease stages, focusing on neutrophils, DC subsets [myeloid (mDCs), plasmacytoid (pDCs)], and monocyte subsets (classical, intermediate, nonclassical). Using flow cytometry, we quantified key innate and adaptive immune populations in patients with active UC, remission, and healthy controls (**Figure 1A**). Relative cell fractions across groups are summarized in **Figure 1B**.

### Elevated neutrophil count in both active and remission phases

**Figure 3:**
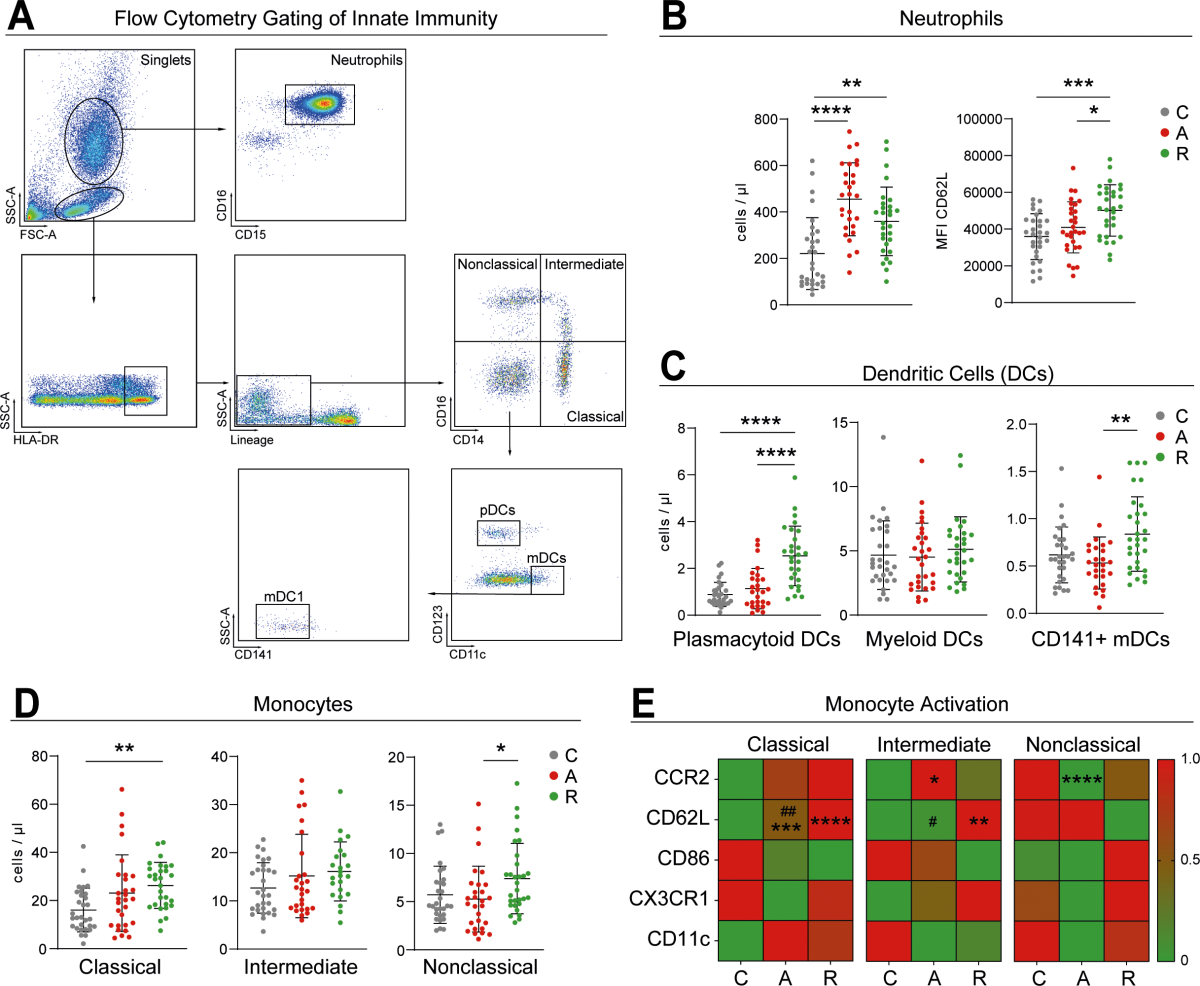
Peripheral innate immune profile. Representative gating strategy for characterization of mononuclear cells and neutrophil granulocytes based on size and granularity. Neutrophils are gated by their expression of CD15 and CD16. Mononuclear cells were gated on HLA-DR+ with lineage exclusion of CD3^+^, CD19^+^, CD56^+^, CD66b^+^ cells. Further gating of monocytes based on CD16 and CD14 expression, identifying classical (CD14^+^, CD16^-^), intermediate (CD14^+^, CD16^+^) and nonclassical (CD14^-^, CD16^+^) monocytes. CD14^-^ and CD16^-^ events were gated based on CD11c and CD123 expression in plasmacytoid dendritic cells and myeloid dendritic cells **(A)**. Neutrophil granulocytes cell count and MFI expression of CD62L are shown **(B)**, along with the cell count of dendritic cell subsets: plasmacytoid dendritic cells, myeloid dendritic cells and CD141^+^ myeloid dendritic cells (CD141^+^mDC) **(C)**. Cell count of monocyte subpopulations including classical, intermediate and nonclassical subsets **(D)**. Heatmap showing an overview of monocyte activation markers per monocyte subpopulation on each study group (C for controls, A for Active and, R for remission). Asterisks show significance between controls and the UC groups, while hash (#) show differences between the UC subgroups **(E)**. All plots portray the mean with standard deviation. Analysis was performed using the one-factorial analysis of variance (ANOVA) for parametric distribution and the Kruskal-Wallis test for non-parametric distribution. Significant differences are indicated by: *#p ≤ 0.05; **##*p* ≤ 0.01; ***p ≤ 0.001; ****p ≤ 0.0001.

Peripheral innate immune cells were analyzed following the gating strategy depicted in **Figure 3A**. Absolute neutrophil cell counts were significantly elevated in active UC, and remained elevated during remission compared to healthy controls (C: 221.1 ± 154.8 cells/µl; A: 454.7 ± 156.9 cells/µl; R: 359.8 ± 147.5 cells/µl). In addition, neutrophils from patients in remission showed significantly higher CD62L expression than those from active UC and controls C: 35920.53 ± 12456.81 Mean fluorescence intensity (MFI); A: 40691.34 ± 14062.42 MFI; R: 50176.87 ± 13961.87 MFI), indicating sustained alterations in trafficking-associated markers (13) (**Figure 3B**).

### Increased DCs subsets during remission

Plasmacytoid DCs were significantly increased during remission compared with both active UC and healthy controls (C: 0.89 ± 0.52 cells/µl; A: 1.14 ± 0.86 cells/µl; R: 2.54 ± 1.27 cells/µl). In contrast, total mDC did not differ across groups (C: 4.67 ± 2.67 cells/µl; A: 4.52 ± 2.64 cells/µl; R: 5.12 ± 2.54 cells/µl). However, within the mDC compartment, CD141+ mDCs were significantly elevated during remission compared to active UC (C: 0.62 ± 0.29 cells/µl; A: 0.53 ± 0.27 cells/µl; R: 0.84 ± 0.39 cells/µl) (**Figure 3C**).

### Elevated monocyte subsets and altered activation profiles in UC

Classical monocytes were significantly elevated in both active UC and remission compared to controls (C: 16.06 ± 9.11 cells/µl; A: 23.07 ± 15.88 cells/µl; R: 26.26 ± 9.54 cells/µl), while nonclassical monocytes were increased in remission (C: 5.71 ± 2.96 cells/µl; A: 5.26 ± 3.42 cells/µl; R: 7.39 ± 3.65 cells/µl). Intermediate monocyte remained unchanged (C: 12.68 ± 5.23 cells/µl; A: 15.18 ± 8.66 cells/µl; R: 16.12 ± 6.12 cells/µl) (**Figure 3D**).

Activation profiles revealed increased CD62L expression in classical (C: 52203.97 ± 19262.13 MFI; A: 67811.28 ± 26418.67 MFI; R: 83456.17 ± 24700.83 MFI) and intermediate (C: 37659.63 ± 16358.91 MFI; A: 38277.24 ± 16212.63 MFI; R: 48283 ± 17137.19 MFI) monocytes during remission. Intermediate monocytes also showed elevated CCR2 during active disease (CCR2: C: 121532.17 ± 21749.56 MFI; A: 131447.41 ± 25430.20 MFI; R: 124537.30 ± 28894.45 MFI), while nonclassical monocytes exhibited lower CCR2 in the active phase (CCR2 C: 59320.37 ± 11988.76 MFI; A: 46532 ± 27133.18 MFI; R: 52915.1 ± 22870.37 MFI). No group differences were detected in CD86, CX3CR1, or CD11c expression (**Figure 3E**).

### T cell subset imbalance in UC

**Figure 4:**
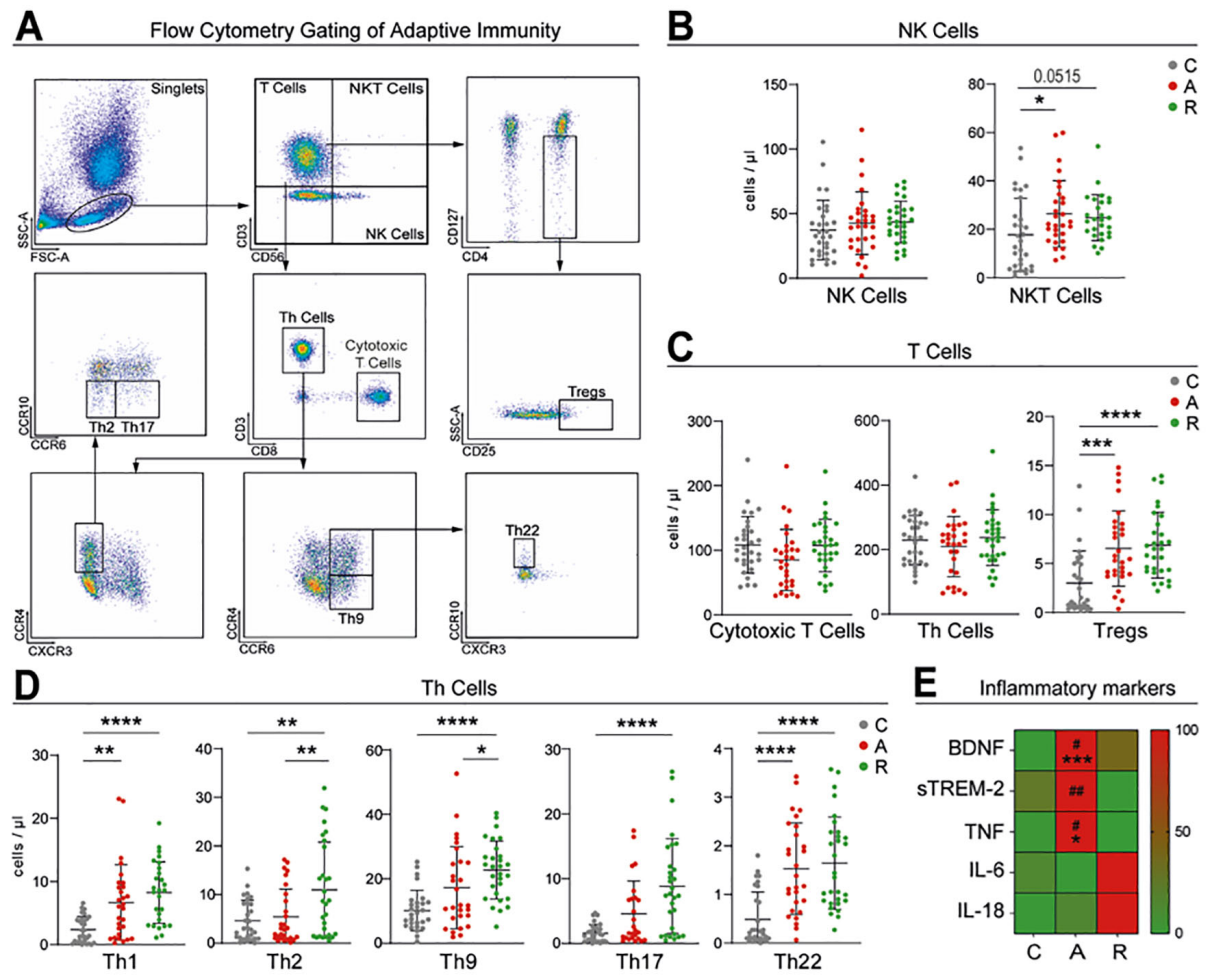
Adaptive immunity mainly focusing on T cells. Representative gating strategy utilized for adaptive immune cells. Singlets were identified by forward (FSC-H) and side scatter (SSC-A). Mononuclear cells were subdivided into T cells (CD3^+^CD56^-^) and natural killer T cells (CD3^+^CD56^+^). Further, T cells were divided into cytotoxic T cells (CD3^+^CD8^+^), T helper cells (CD3^+^CD4^+^) and regulatory T cells (CD3^+^CD4^+^CD25^+^CD127^-^). The T helper cells were divided into different subpopulations: T helper 1 cells (CD4^+^CXCR3^+^), T helper 2 cells (CD4^+^CCR4^+^CCR6^-^), T helper 9 cells (CD4^+^CCR4^-^CCR6^+^), T helper 17 cells (CD4^+^CCR4^+^CCR6^+^) and T helper 22 cells (CD4^+^CCR4^+^CCR6^+^CCR10^+^) **(A)**. Scatter dot plots illustrating the cell counts of natural killer cells and natural killer T cells **(B)**, as well as cytotoxic T cells, T helper cells, and regulatory T cells **(C)**. Overview of the T helper subpopulations: T helper 1 cells, T helper 2 cells, T helper 9 cells, T helper 17 cells and T helper 22 cells **(D)**. Heatmap visualizing the normalized concentration of soluble plasma markers (BDNF, sTREM-2, TNF, IL-6, IL-18). Asterisks show significance between controls and the UC groups, while hash (#) show differences between the UC subgroups **(E)**. All graphs are showing mean value and standard deviation. The groups were analyzed using the one-factorial analysis of variance (ANOVA) for parametric distribution and the Kruskal-Wallis test for non-parametric distribution. Significant differences are indicated by: *#p ≤ 0.05; **##*p* ≤ 0.01; ***p ≤ 0.001; ****p ≤ 0.0001.

We next assessed adaptive immune cell profiles across disease stages (**Figure 4A**). While CD4+ Th and CD8+ cytotoxic T cells remained unchanged, NKT cells were elevated in active UC and remained increased during remission (C: 17.77 ± 15.13 cells/µl; A: 26.45 ± 13.73 cells/µl; R: 24.77 ± 9.50 cells/µl). Tregs were also significantly increased in both UC groups compared to controls (C: 3.01 ± 3.28 cells/µl; A: 6.54 ± 3.86 cells/µl; R: 6.88 ± 3.36 cells/µl) (**Figures 4B, C**).

Within the Th compartment, Th17 cells were elevated in remission (C: 1.57 ± 1.37 cells/µl; A: 4.56 ± 5.07 cells/µl; R: 8.83 ± 7.32 cells/µl), while Th2 and Th9 cells increased significantly in remission (Th2: C: 4.63 ± 4.20 cells/µl; A: 5.40 ± 5.72 cells/µl; R: 10.98 ± 9.84 cells/µl; Th9: C: 10.20 ± 6.26 cells/µl; A: 17.27 ± 12.76 cells/µl; R: 22.72 ± 9.02 cells/µl). Th1 and Th22 cell counts were significantly increased in both active UC and remission (Th1: C: 2.40 ± 2.06 cells/µl; A: 6.69 ± 5.97 cells/µl; R: 8.25 ± 4.85 cells/µl; Th22: C: 0.49 ± 0.56 cells/µl; A: 1.53 ± 0.94 cells/µl; R: 1.64 ± 0.95 cells/µl). Overall, all Th subsets were found significantly elevated in remission (**Figure 4D**).

### Elevated plasma inflammatory markers during active UC

To assess systemic inflammation, we measured peripheral levels of BDNF, sTREM-2, TNF, IL-6, and IL-18. These cytokines and neuroimmune markers have been linked to mood symptoms and gut-brain axis disruption in IBD (14). BDNF was significantly elevated during active UC (C: 407.53 ± 235.08 pg/mL; A: 838.32 ± 462.82 pg/mL; R: 574.43 ± 240.14 pg/mL), as was sTREM-2, particularly compared to remission (C: 121.69 ± 113.24 pg/mL; A: 263.18 ± 236.84 pg/mL; R: 72.80 ± 70.58 pg/mL). TNF levels followed a similar pattern, peaking in active disease (C: 38.59 ± 21.14 pg/mL; A: 58.13 ± 30.69 pg/mL; R: 38.12 ± 19.04 pg/mL). While IL-6 and IL-18 did not differ significantly, trends toward increased levels in remission were observed (**Figure 4E**).

### Sex-specific differences in immune responses

**Figure 5:**
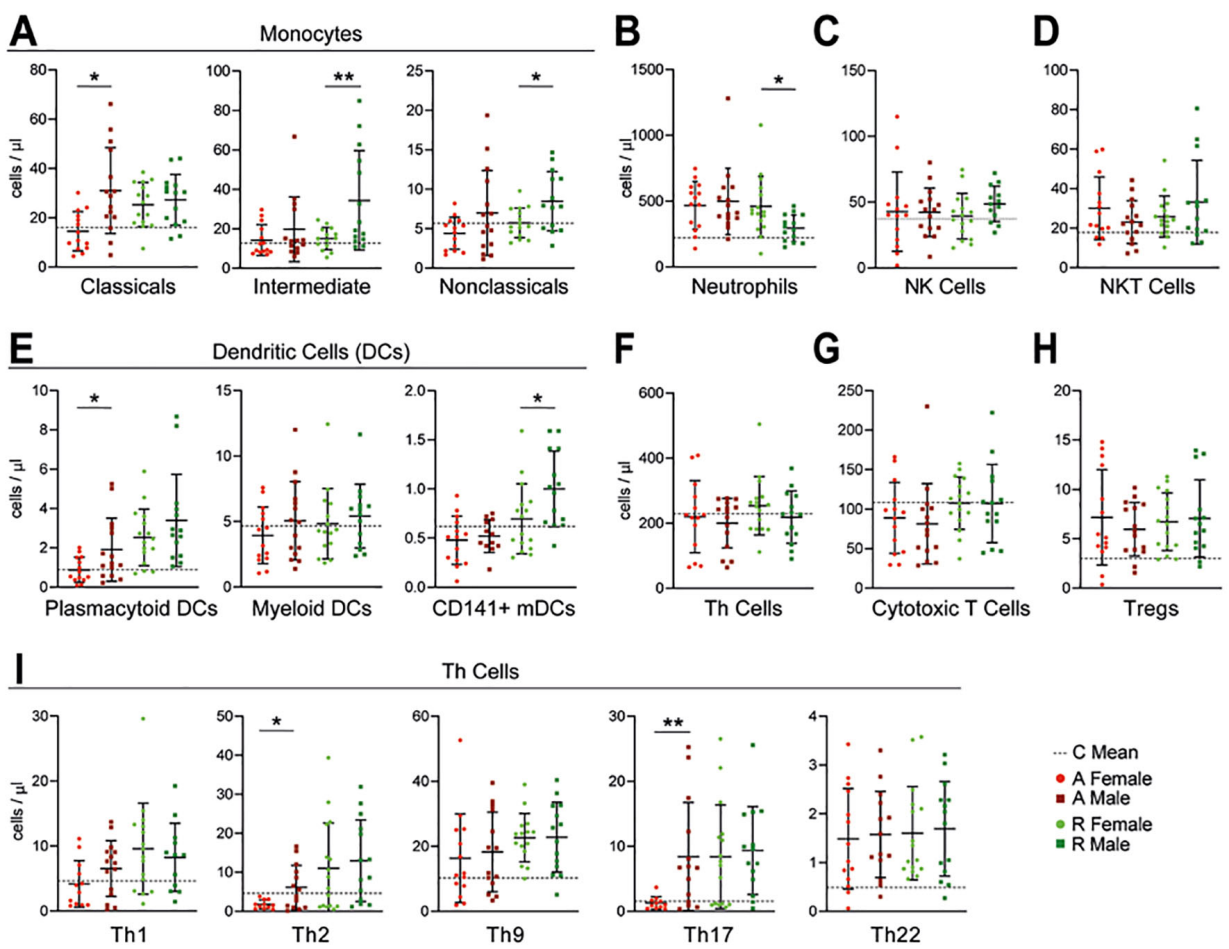
Sex differences in immune cell subsets. Cell counts of different immune cell subsets based on sex: monocytes **(A)**, neutrophils **(B)**, natural killer cells **(C)**, natural killer T cells **(D)**, dendritic cells **(E)**, T helper cells **(F)**, cytotoxic T cells **(G)**, regulatory T cells **(H)** and T helper cell subsets **(I)**. Lighter circles indicate female participants and darker squares indicate males. All graphs are showing mean value and standard deviation, dotted lines represent the mean of controls according to the shown cell type. The groups were analyzed using the unpaired t-test for parametric distribution and Mann-Whitney-U test for non-parametric distribution. Significant differences are indicated by: *p ≤ 0.05; **p ≤ 0.01.

UC is known to differ between sexes in presentation, treatment, comorbidities, and quality of life (10, 15). To assess sex-related immune differences, we analyzed innate and adaptive immune cells by sex across disease stages. During active UC, male patients showed higher total monocyte counts than females (A: ♀: 14.55 ± 7.95 cells/µl, ♂: 31.03 ± 17.46 cells/µl), with elevated intermediate (R: ♀: 15.11 ± 5.62 cells/µl, ♂: 34.42 ± 25.22 cells/µl) and nonclassical monocytes (R: ♀: 5.73 ± 1.89 cells/µl, ♂: 8.47 ± 3.74 cells/µl) during remission (**Figure 5A**). Neutrophil levels were similar in both sexes during active UC, but males showed lower counts in remission (R: ♀: 460.7 ± 227.3 cells/µl, ♂: 295.9 ± 100.2 cells/µl) (**Figure 5B**). DC subset analysis revealed increased pDCs in males during active disease (A: ♀: 0.88 ± 0.64 cells/µl, ♂: 1.91 ± 1.61 cells/µl) and CD141+ mDCs in remission (R: ♀: 0.70 ± 0.36 cells/µl, ♂: 1.00 ± 0.39 cells/µl) (**Figure 5E**).

Although total Th cell counts did not differ by sex, males exhibited significantly elevated Th2 and Th17 subsets in active UC (Th2: A: ♀: 1.76 ± 1.12 cells/µl, ♂: 6.13 ± 5.65 cells/µl; Th17:A: ♀: 1.28 ± 0.97 cells/µl, ♂: 8.42 ± 8.31 cells/µl) (**Figure 5I**). No sex differences were observed in NK, CD8+ T, NKT, or Treg populations (**Figures 5C,D, F–H**), nor in cytokine levels (**Supplementary Figure 1**).

### Severe fatigue is more prevalent during active UC

**Figure 6:**
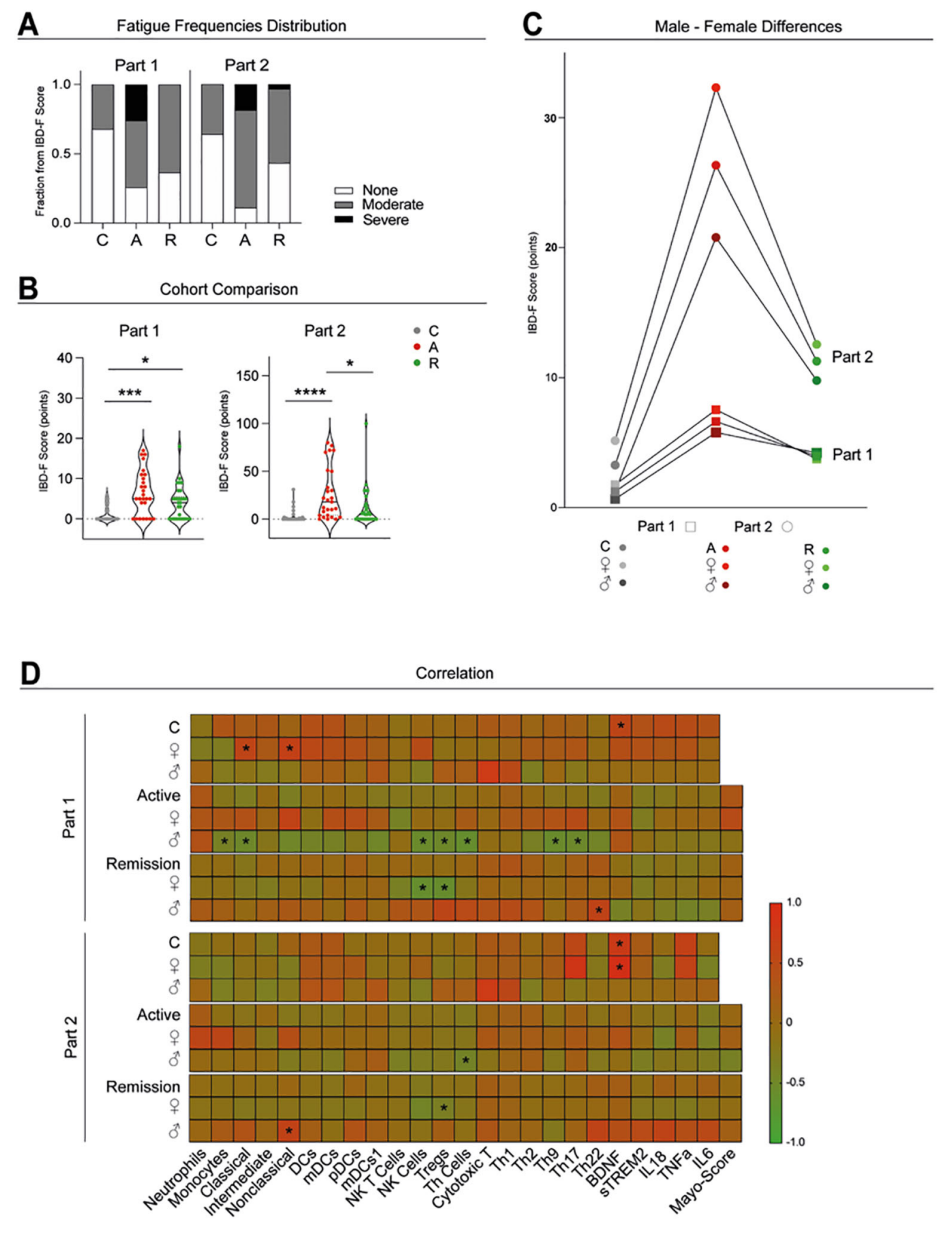
Fatigue assessment and correlation to immune cell parameters. Fatigue Frequency Distribution of the three categories none, mild and severe fatigue within the two parts of the IBD-F questionnaire in the three study groups: active (A), remission (R) and healthy control (C) **(A)**. Violin plots showing individual IBD-F questionnaire values for part 1 and 2 **(B)**. Fatigue Score differences between male and female participants in the three groups: A, R and C **(C)**. Heatmap showing a correlation between the two parts of the IBD-F and previously assessed immune cell parameters and soluble plasma markers **(D)**. For **(A–C)** plot, analysis was performed using one-factorial analysis of variance (ANOVA) for parametric distribution and the Kruskal-Wallis test for non-parametric distribution. For **(D)**, non-parametric Spearman Correlation was used to correlate fatigue with immune cells, soluble markers and demographic data. Significant differences are indicated as: *p ≤ 0.05; ***p ≤ 0.001; ****p ≤ 0.0001.

Fatigue was assessed using the IBD-F questionnaire to evaluate prevalence and severity across groups and sexes. Part 1 of the questionnaire showed that severe fatigue was exclusive to active UC (None: C: 68,0%; A: 25,9%; R: 36,7%; Moderate: C: 32,0%; A: 48,1%; R: 63,33%; Severe: C: 0%; A: 25,9%; R: 0%). Part 2 revealed a similar trend (None: C: 64,0%; A: 11,11%; R: 43,33%, Moderate: C: 36,00%; A: 70,37%; R: 53,33%; Severe: C: 0%; A: 18,52%; R: 3,33%) (**Figure 6A**).

Total fatigue scores were significantly higher in UC patients, especially during active disease (Part 1: C: 1.24 ± 2.01 points, A: 6.40 ± 5.51 points, R: 3.97 ± 4.23 points; Part 2: C: 3.28 ± 7.36 points, A: 25.39 ± 27.04 points, R: 11.27 ± 20.06 points) (**Figure 6B**). Females generally reported higher fatigue scores than males, except in Part 1 during remission, where males reported slightly higher scores (♂ R: 4.21± 3.64 points; ♀R: 3.75 ± 4.80 points) (**Figure 6C**).

### Sex-specific correlations between fatigue and immune cell profiles

Fatigue scores were correlated with immune cell populations and inflammatory markers in a sex- stratified manner. In IBD-F Part 1, plasma BDNF levels positively correlated with fatigue in (C: r = 0.62). In female controls, fatigue scores also showed positive correlations with classical and nonclassical monocytes (both C: ♀: r = 0.57). During active UC, males showed strong negative correlations between fatigue and classical monocytes (A: ♂: r = -0.57), NK cells (A: ♂: r = -0.56), Tregs (A: ♂: r = -0.58), Th9 (A: ♂: r = -0.71), and Th17 (A: ♂: r = -0.58). In remission, fatigue in females inversely correlated with NK cells (R: ♀: r = -0.61) and Tregs (R: ♀: r = -0.74), while males showed a positive correlation with Th22 cells (R: ♂: r = 0.6).

In IBD-F Part 2, BDNF again correlated with fatigue in controls (C: ♂: r = 0.54, C: ♀: r = 0.85). During active UC, Th cells negatively correlated with fatigue in males (A: ♂: r = -0.58). In remission, Th22 cells correlate positively with fatigue (C: r = 0.39), while female fatigue again inversely correlated with Tregs (R: ♀: r = -0.56). Males showed a positive correlation with nonclassical monocytes whereas males showed a positive correlation of fatigue with nonclassical monocytes (R: ♀: r = 0.62) (**Figure 6D**). Given the exploratory nature of these analyses, uncorrected p-values are presented; results should be interpreted as hypothesis-generating.

## Discussion

Ulcerative colitis is marked by recurrent inflammation and immune dysfunction, but how peripheral immunity reflects disease activity and symptom burden remains unclear. By profiling peripheral blood across disease stages, our study uncovers distinct shifts in neutrophils, dendritic cells, monocyte subsets, and T cell populations that mirror changes in disease activity and immune tone. We further show that fatigue severity aligns with specific immune signatures and varies by sex, emphasizing the importance of considering sex-based immune differences in efforts to refine and personalize UC treatment.

The innate immune compartment showed clear signatures of disease activity, most prominently an increase in circulating neutrophils during active UC. Neutrophils were significantly elevated in both active and remission phases, consistent with their well-established role in UC pathogenesis and potential biomarkers of disease severity (16). In active UC, enhanced neutrophil counts likely reflect acute inflammatory responses, aligning with previous reports linking neutrophil infiltration to disease severity, tissue damage, and relapse risk (17). Neutrophils contribute to gut injury through secretion of TNF, IL-1β, MPO, and NETs, exacerbating epithelial barrier dysfunction. However, their persistent elevation during remission could reflect a more complex role potentially involving tissue repair or low-grade, unresolved inflammation. We observed increased CD62L expression on neutrophils during remission, which may reflect ongoing tissue homing and repair processes. CD62L facilitates leukocyte trafficking, and its upregulation has been proposed as a predictor of treatment response (18), supporting its potential relevance as both biomarker and therapeutic target.

DCs and monocytes displayed distinct alterations across disease stages, consistent with their roles in both inflammation and resolution. During remission, pDCs and CD141+ myeloid DCs were selectively expanded, driving a shift toward immune modulation and resolution as previously suggested by Gren & Grip, et al. (19) and Sun et al. (20). These subsets contribute to mucosal healing by producing IL-10 and type I interferons, promoting regulatory T cell induction and curbing proinflammatory responses (21). In parallel, monocyte dynamics reflected a complementary shift in immune tone. Classical and nonclassical monocytes were increased in remission, accompanied by higher expression of CD62L and CCR2, molecules central to immune cell recruitment. This expression pattern may reflect a functional shift from inflammatory infiltration to tissue repair. Targeting CCR2 has shown therapeutic benefit in colitis by reducing macrophage infiltration and cytokine release (22), and CD62L has emerged as a promising predictor of infliximab response (18), suggesting these pathways may hold translational relevance. Additionally, monocyte-mediated efferocytosis, essential for clearing apoptotic neutrophils, may support the resolution of inflammation and reinforce their dual role in UC pathophysiology (23).

We next explored T cell dynamics across UC stages. In active disease, elevated Th1 and Th17 cells likely perpetuate inflammation through IFN-γ and IL-17 signaling, disrupting epithelial integrity and sustaining immune activation (24, 25). These findings are consistent with prior reports linking Th17 responses to both mucosal injury and systemic immune dysregulation in UC (26). Interestingly, Th17 and Th1 cells remained elevated during remission, suggesting ongoing low-grade inflammation despite clinical quiescence. This persistent effector T cell activity could reflect an immunological vulnerability, potentially contributing to relapse risk or chronic symptoms. These findings suggest that Th17/Treg profiling may serve as an immunological indictor of remission quality.

Tregs, increased in both active disease and remission, likely act to counterbalance proinflammatory signals. Their upregulation during remission aligns with a compensatory role in restoring immune homeostasis, however sustained elevation could also indicate a failure to fully resolve inflammation (27, 28). Similarly, increases in Th9 and Th22 cells during remission may reflect tissue-repair mechanisms, since Th9 cells promote mast cell activation and DC recruitment, while Th22 cells support epithelial regeneration via mucin and antimicrobial peptide production (29, 30).

Overall, our findings suggest an imbalance between pro-inflammatory and regulatory Th cell responses. These results raise the possibility that Th subset profiling could serve as a biomarker for disease activity or remission stability, given the distinct roles of individual T cell populations. This aligns with ongoing efforts to explore Treg-based therapies in refractory UC (31), highlighting the broader interest in targeting Th subsets for precision immunotherapy.

Beyond gut-localized inflammation, UC is increasingly recognized as a systemic disorder with CNS manifestations, including fatigue and mood disturbances (11, 32, 33). Consistent with this view, fatigue in UC has been shown to correlate more strongly with depressive symptoms and younger age than with anemia or objective measures of disease activity and is not reliably associated with CRP as a marker of systemic inflammation, underscoring that fatigue cannot be explained solely by inflammatory burden or biological disease activity (34). In our study, we observed elevated peripheral levels of BDNF, sTREM-2, and TNF during active disease, suggesting involvement of neuroimmune signaling pathways, as TNF and BDNF levels correlated with fatigue severity, especially in the active phase (6, 35–39). These molecules influence neuronal plasticity, microglial activation, and blood-brain barrier integrity, providing a mechanistic bridge between inflammation and CNS symptoms. Persistent immune activity in remission, particularly involving neutrophils and monocytes, may sustain systemic inflammatory signaling and contribute to chronic fatigue via altered cytokine production and neuroimmune crosstalk (40). The positive effects of anti-TNF and IL-inhibitors on fatigue support this model and point toward the need for therapies that restore immune balance beyond clinical remission (41–43). Taken together, fatigue may not simply reflect disease activity but rather unresolved immune dysregulation deserving consideration as a functional marker of systemic inflammation in IBD.

Sex differences have emerged as a critical determinant of immune responses in UC, though their underlying mechanisms remain incompletely understood. Our study provides the first evidence of a sex-specific increase in Th cell subsets in males with active UC. Males exhibited elevated monocyte and DC counts alongside increased Th2 and Th17 cells during active disease, suggesting a heightened proinflammatory phenotype. These shifts may be hormonally mediated, with testosterone promoting Th1/Th17 polarization (44), potentially contributing to the more aggressive disease course observed in men. Interestingly, we observed a Th2-dominant profile in males, challenging the traditional view of stronger Th2 responses in females and suggesting sex-specific immune dynamics in UC (45). Conversely, females displayed persistent neutrophil levels during remission, possibly driven by estrogen-mediated effects on neutrophil migration and cytokine responsiveness (46). This may reflect subclinical inflammation and could be one factor contributing to the greater fatigue burden reported by women. Notably, sex also shaped fatigue–immune correlations. In males, fatigue inversely correlated with Th9 and Th17 cell counts, while in females, it was linked to innate immune cell dysregulation, including NK cells and monocytes. Elevated TNF and IL-6 levels in males may indicate stronger responsiveness to anti-TNF therapies, whereas persistent neutrophil activity in females during remission highlights the importance of monitoring subclinical inflammation to prevent relapse (47, 48).

Taken together, our findings position fatigue as a symptom with potential utility for identifying peripheral immune dysregulation in UC. Persistent increased neutrophils and monocytes, along with imbalanced T cell responses, likely contributes to systemic inflammation and neuroimmune crosstalk. The sex-specific fatigue–immune correlations we observed underscore distinct biological drivers across sexes. Altogether, fatigue emerges not just as a symptom, but as a potential clinical marker of unresolved immune activity.

This study provides a comprehensive peripheral immune map across UC disease stages, linking innate and adaptive immune shifts to fatigue and sex-based immune modulation. We demonstrate that neutrophils and DCs exhibit distinct activity patterns across disease phases, with adaptive T cell profiles suggesting ongoing immune remodeling during remission. Elevated cytokines associated with neuroimmune signaling support the hypothesis that UC-related inflammation extends beyond the gut. We present novel evidence of sex-specific T helper cell alterations in active disease, with male patients displaying a proinflammatory Th17/Th2 signature. These differences may influence treatment efficacy, particularly for biologics targeting TNF or IL-23 pathways.

Our findings suggest that peripheral immune profiling, including T cell subsets and monocyte marker analysis, could enhance disease monitoring and fatigue risk stratification in UC. Future longitudinal studies are needed to validate these signatures and explore their predictive utility for relapse, therapy response, and long-term symptom burden.

### Strengths and limitations

Key strengths of this study include the comparison of two UC disease stages with a matched healthy control group, allowing for a detailed analysis of immune alterations across disease progression. To our knowledge, this is the first study to comprehensively examine both innate and adaptive immune profiles in UC in combination with cytokine measurements, validated fatigue assessments, and sex-specific immune analyses in this overview. Crucially, we link immune signatures to fatigue severity which is a symptom often underexplored in UC, while minimizing confounding through sex- and age-matched controls and the exclusion of individuals with neurodegenerative or psychiatric comorbidities. This yielded a well-defined cohort suitable for investigating neuroimmune and fatigue-related mechanisms in UC.

Limitations include the cross-sectional design, which limits assessment of longitudinal immune changes, and a predominantly Caucasian, Germany-based cohort, restricting broader generalizability. Additionally, remission classification relied on clinical and biochemical criteria (partial Mayo score ≤2 and fecal calprotectin <250 µg/g) without endoscopic confirmation. The stricter STRIDE-II threshold of <100 µg/g for deep mucosal healing was not applied, and residual mucosal inflammation in some remission patients cannot be excluded. No a priori power calculation was performed, as sample size was determined by recruitment availability; this limits the statistical power of the analyses, particularly for sex-stratified subgroups. The sex-stratified correlation analyses were exploratory in nature, given the relatively small subgroup sizes, and findings should be interpreted accordingly. Routine laboratory parameters including hemoglobin and ferritin were available for a subset of UC patients. A minority met clinical criteria for anemia (active: n=3/29; remission: n=2/30). Iron deficiency, assessed by serum ferritin (<30 ng/ml), was more prevalent in active UC (n=11/29) than in remission (n=3/30), and cannot be fully excluded as a contributing factor to fatigue severity in the active group. Data on thyroid function and vitamin deficiencies were not systematically collected and are not available for this cohort. Treatment varied considerably, encompassing biologics, JAK inhibitors, and aminosalicylates in diverse combinations, precluding formal statistical correction for medication as a covariate. While treatment effects on peripheral immune profiles cannot be fully excluded, the observed alterations are broadly consistent with the existing literature, supporting their biological plausibility. Future longitudinal studies with larger, treatment-stratified cohorts are needed to validate these immune signatures and clarify the relative contributions of disease activity and therapy.

## Data Availability

The original contributions presented in the study are included in the article/**Supplementary Material**. Further inquiries can be directed to the corresponding author.

## Supplementary material

Supplementary Figure 1 Sex differences in cytokine levels. Cytokine levels (BDNF, sTREM2, TNF, IL-6, IL-18) were analyzed based on male/female subgroups. The groups were analyzed using the unpaired t-test for parametric distribution and Mann-Whitney-U test for non-parametric distribution.
