# Supplementary figures and images for: Sex-related peripheral immune profile in ulcerative colitis: links to fatigue
Source: Front Immunol. 2026 May 14;17:1824822. doi: 10.3389/fimmu.2026.1824822 (PMC13215802; doi:10.3389/fimmu.2026.1824822)

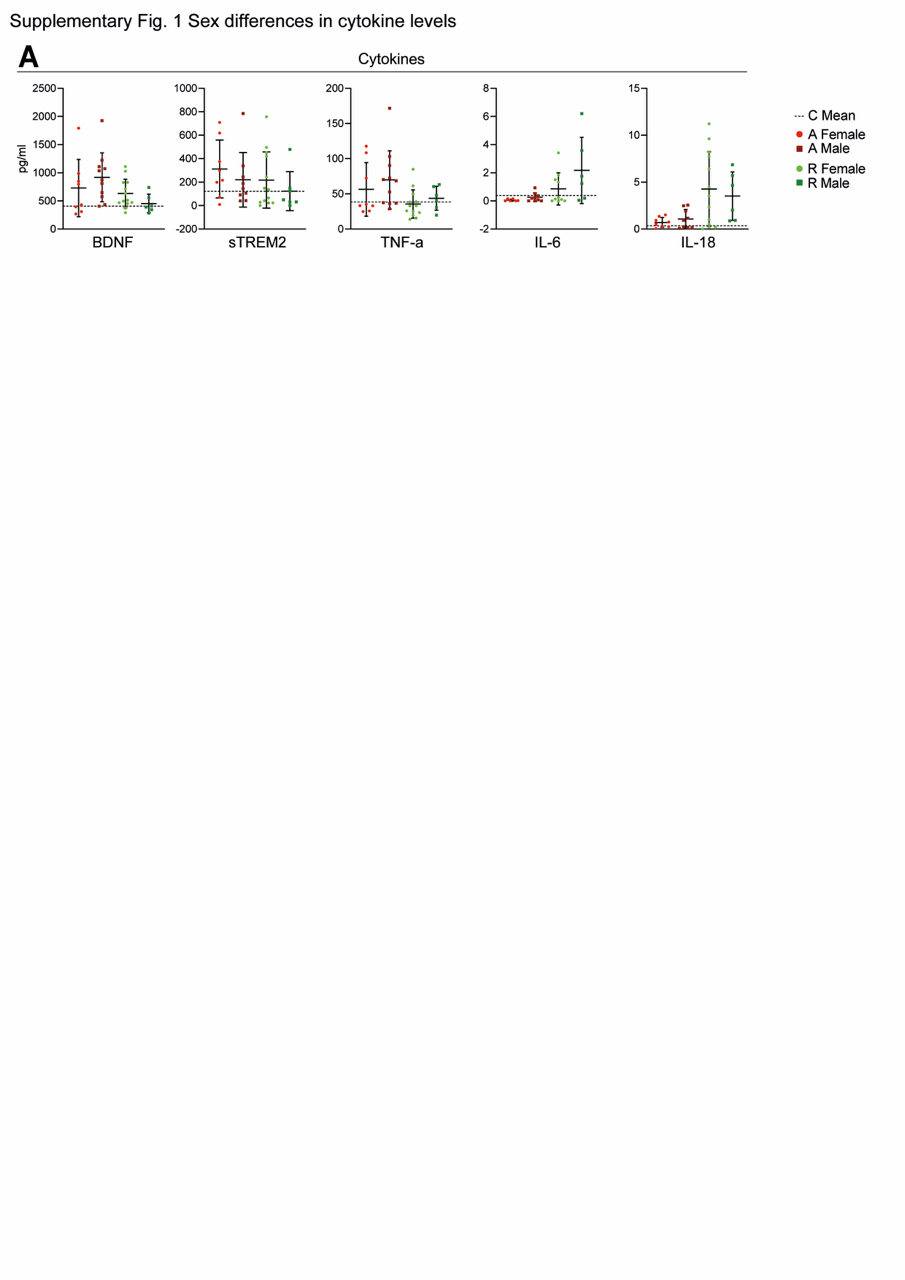

Supplement: Supplementary Figure 1 — Sex differences in cytokine levels. Cytokine levels (BDNF, sTREM2, TNF, IL-6, IL-18) were analyzed based on male/female subgroups. The groups were analyzed using the unpaired t-test for parametric distribution and Mann-Whitney-U test for non-parametric distribution. [file Image1.tiff]
