# Supplementary material for: Sex-related peripheral immune profile in ulcerative colitis: links to fatigue
Source: Front Immunol. 2026 May 14;17:1824822. doi: 10.3389/fimmu.2026.1824822 (PMC13215802; doi:10.3389/fimmu.2026.1824822)
Supplement: Supplementary file 2 [file Table1.docx]

| **Supplementary Table 1: ANCOVA results for group comparisons after adjustment for age, sex, and BMI** | | | | | |  |  |  |
| --- | --- | --- | --- | --- | --- | --- | --- | --- |
| *Variable* | *n* | *F* | *p* | *Significance* | *Survives*  *adjustment* | *Active vs Controls* | *Remission vs Controls* | *Active vs Remission* |
| Neutrophils | 89 | 13.339 | 0 | *** | Yes | *** | ** | ns |
| CD62L on neutrophils (MFI) | 89 | 7.487 | 0.001 | ** | Yes | ns | ** | * |
| pDCs | 89 | 15.408 | 0 | *** | Yes | ns | *** | *** |
| CD141+ mDCs | 89 | 4.251 | 0.0176 | * | Yes | ns | ns | * |
| Classical monocytes | 89 | 4.495 | 0.0141 | * | Yes | ns | ** | ns |
| Nonclassical monocytes | 89 | 1.086 | 0.3424 | **ns** | **No** | **ns** | **ns** | **ns** |
| CD62L on classical monocytes (MFI) | 89 | 13.686 | 0 | *** | Yes | * | *** | * |
| CD62L on intermediate monocytes (MFI) | 89 | 4.878 | 0.01 | * | Yes | ns | ** | ns |
| CCR2 on intermediate monocytes (MFI) | 89 | 1.427 | 0.2461 | **ns** | **No** | **ns** | **ns** | **ns** |
| CCR2 on nonclassical monocytes (MFI) | 89 | 2.5 | 0.0885 | **ns** | **No** | **ns** | **ns** | **ns** |
| NKT cells | 89 | 3.292 | 0.0423 | * | Yes | ns | * | ns |
| Tregs | 89 | 5.681 | 0.0049 | ** | Yes | ns | ** | ns |
| Th1 cells | 89 | 7.069 | 0.0015 | ** | Yes | ns | ** | ns |
| Th2 cells | 89 | 4.392 | 0.0155 | * | Yes | ns | * | * |
| Th17 cells | 89 | 7.069 | 0.0015 | ** | Yes | ns | ** | ns |
| Th9 cells | 89 | 5.041 | 0.0087 | ** | Yes | ns | ** | ns |
| Th22 cells | 89 | 4.23 | 0.0179 | * | Yes | * | ns | ns |
| BDNF | 89 | 3.617 | 0.0341 | * | Yes | * | ns | ns |
| sTREM-2 | 89 | 0.701 | 0.5009 | **ns** | **No** | **ns** | **ns** | **ns** |
| TNF | 89 | 1.683 | 0.1961 | **ns** | **No** | **ns** | **ns** | **ns** |

* p<0.05, ** p<0.01, *** p<0.001, ns = not significant

ANCOVA model: outcome ~ Group + Age + Sex + BMI

Post-hoc pairwise comparisons performed using Tukey HSD on covariate-adjusted values
