# Supplementary material for: Sex-related peripheral immune profile in ulcerative colitis: links to fatigue
Source: Front Immunol. 2026 May 14;17:1824822. doi: 10.3389/fimmu.2026.1824822 (PMC13215802; doi:10.3389/fimmu.2026.1824822)
Supplement: Supplementary file 3 [file DataSheet1.docx]

**Supplementary Methods**

This file provides detailed description of the experimental procedures, assays and statistical analyses used on our study.

**Study design and participants**

The present study cohort comprises 89 participants categorized into three groups: active UC (n=29), UC in remission (n=30) and healthy controls (n=30). Participants were enrolled from January to October 2021, at the outpatient clinic of the University Clinic for Gastroenterology, Hepatology and Infectiology at Otto von Guericke University Magdeburg. The healthy controls were recruited from staff and medical students at the Otto von Guericke University Magdeburg and were matched on average age and sex distribution to the UC cohort (Fig. 1A). A detailed demographic overview is provided in Table 1.

All enrolled participants were ≥ 18 years of age, with a diagnosis of UC based on clinical manifestations, endoscopic and imaging features or pathological findings according to the European Crohn’s and Colitis Organisation (ECCO) consensus guidelines(1), able to give written informed consent and did not meet any of the following exclusion criteria: antibiotic use in the last 3 months, serious complications of UC (e.g. toxic megacolon, perforation, fistules), oncologic diseases, Alzheimer’s disease, Parkinson’s disease, Multiple Sclerosis, Amyotrophic Lateral Sclerosis, history of stroke, Crohn’s disease, Celiac disease, depression, anxiety, schizophrenia, autism, diabetes, and unstable or uncontrolled asthma, COPD and arterial hypertension.

Classification into active and remission groups was based on the partial Mayo score (2,3) and fecal calprotectin. The partial Mayo Score assess disease activity and include the following sub-items: stool frequency, rectal blood discharge and physician's global assessment (0 to 3 points for each item). Active UC was defined as a partial Mayo score >2 and fecal calprotectin >250 µg/g; remission was defined as a partial Mayo score ≤2 and calprotectin <250 µg/g. This threshold reflects real-world clinical practice at the time of recruitment; the stricter STRIDE-II target of <100 µg/g for deep mucosal healing was not applied. Calprotectin values were available for a subset of participants (active: n=21/29, mean 880.32 ± 721.52 µg/g; remission: n=18/30, mean 161.33 ± 234.55 µg/g), indicating appropriate group separation overall, though heterogeneity within the remission group means residual mucosal inflammation cannot be fully excluded in all patients. Among UC patients, a minority met clinical criteria for anemia (active: n=3/29; remission: n=2/30). Iron deficiency, assessed by serum ferritin, was more prevalent in active UC (n=11/29) than in remission (n=3/30), and cannot be fully excluded as a contributing factor to fatigue severity in the active group. Our study was conducted in accordance with the ethical principles for the Declaration of Helsinki with approval from the ethics committee of the Medical Faculty, Otto-von-Guericke Magdeburg (No 190/21).

**Whole blood lysis and staining**

In this study, a venous blood sample of 4 mL of blood was collected into a sterile BD Ethylenediaminetetraacetic acid (EDTA) tube containing 1.8 mg EDTA per milliliter of blood. The whole blood samples were lysed using 1x Red Blood Cell Lysis Buffer (Bio Legend, 10x) following the manufacturer's instructions to remove the erythrocytes from the sample. Afterward, samples were centrifuged at 400 *g* for 5 min at 18°C and supernatant was discarded to remove cell debris. Cell pellet was resuspended in 4 mL Phosphate-buffered saline (PBS) and centrifuged under the same conditions. Cells were further resuspended in 300 μL FACS Buffer (1X PBS, 2–5% Stain Buffer (FBS), 2 mM EDTA and 2 mM NaN_3_) and 100 μL were added to a 5 mL FACS tube to continue with the staining protocol. To prevent non-specific binding of the immune cells to Fc-receptors, the samples were incubated with 5 μL of Human TruStain FcX (BioLegend) for 5min. Each sample was stained and incubated for 30 min at 4°C with the following conjugated antibodies: anti-human CD16 (Fluorescein-5-isothiocyanat), anti-human HLA-DR (Peridinin chlorophyll protein-Cyanine5.5), anti-human CD86 (Allophycocyanin) anti-human CD3 (Alexa Fluor 700), anti-human CD19 (Alexa Fluor 700), anti-human CD66b (Alexa Fluor 700), anti-human CD56 (Alexa Fluor 700), anti-human CD14 (Brilliant Violet 421), anti-human CX3CR1 (Brilliant Violet 510), anti-human CD141 (Brilliant Violet 605), anti-human CD11c (Brilliant Violet 711), anti-human CCR2 (Phycoerythrin), anti-human CD62L (Phycoerythrin -Dazzle 594), anti-human CD15 (Phycoerythrin-Cyanine 5), anti-human CD123 (Phycoerythrin-Cyanine 7), anti-human CD45 (Fluorescein-5-isothiocyanat), anti-human CD25 (Peridinin chlorophyll protein-Cyanine 5.5), anti-human CCR4 (Alexa Fluor 647), anti-human CD56 (Alexa Fluor 700), anti-human CD4 (Allophycocyanin-Cyanine 7), anti-human CD3 (Brilliant Violet 510), anti-human CD127 (Brilliant Violet 605), anti-human CCR6 (Brilliant Violet 711), anti-human CCR10 (Phycoerythrin), anti-human CD8 (Phycoerythrin -Dazzle 594), anti-human CXCR3 (Phycoerythrin-Cyanine 5), anti-human CD19 (Phycoerythrin-Cyanine 7).

The immune cell suspension was washed twice and resuspended in a final volume of 210 µL FACS Buffer. Samples were acquired on the Attune NxT Flow Cytometer (Thermo Fisher Scientific) and analyzed with the FlowJo Analysis Software (v10.3). Fluorescence minus one FMO controls (FMO) were used to assess the background fluorescence and thus correctly distinguish positive and negative populations.

**Flow cytometric gating strategy**

Singlets were identified by forward (FSC-H) and side scatter (SSC-A). Mononuclear cells and granulocytes were identified based on size and granularity; neutrophils were gated by their expression of CD15 and CD16. Mononuclear cells were gated on HLA-DR+ with lineage exclusion of CD3^+^, CD19^+^, CD56^+^, CD66b^+^ cells. The focus of our analysis of innate immune analysis was the characterization and activation of monocytes (HLA-DR^+^CD3^-^CD19^-^CD56^-^CD66b^-^) and dendritic cells (HLA-DR^+^CD3^-^CD19^-^CD56^-^CD66b^-^CD14^-^CD16^-^). Monocytes were categorized into classical (CD14^+^CD16), intermediate (CD14^+^CD16^+^) and nonclassical (CD14^-^CD16^+^) monocytes(4). Dendritic cells were divided into subgroups and identified as myeloid (CD123^-^CD11c^+^) and plasmacytoid (CD123^+^CD11c^-^) dendritic cells (Fig. 2A).

For adaptive immune analyses focussing on T cells, singlets were identified as described above, and mononuclear cells were subdivided into T cells (CD3⁺CD56⁻) and natural killer T cells (CD3⁺CD56⁺). T cells were further categorized into cytotoxic T cells (CD3⁺CD8⁺), T helper (Th) cells (CD3⁺CD4⁺), and regulatory T cells (Tregs; CD3⁺CD4⁺CD25⁺CD127⁻). Th cells were identified as Th cell subsets: Th1 (CD4^+^CXCR3^+^), Th2 (CD4^+^CCR4^+^CCR6^-^), Th9 (CD4^+^CCR4^-^CCR6^+^), Th17 (CD4^+^CCR4^+^CCR6^+^) and Th22 (CD4^+^CCR4^+^CCR6^+^CCR10^+^) (5) (Fig. 3A).

**Blood plasma collection and bead-based cytokine assay**

To obtain plasma from EDTA-treated blood samples, density gradient centrifugation was initially performed. Blood was diluted at a 1:2 ratio with Dulbecco's Phosphate Buffered Saline (DPBS) and layered onto 3 mL of Ficoll. The samples were then centrifuged at 400 *g* for 30 minutes at 18°C, with an acceleration setting of 9 and deceleration set to 0. This process separated the blood into distinct layers within the tube, organized from bottom to top as follows: erythrocytes, granulocytes, Ficoll reagent, lymphocyte layer containing peripheral blood mononuclear cells (PBMCs: lymphocytes and monocytes), and blood plasma. Plasma was then carefully collected via pipette and stored at -80°C until analysis of neuroinflammatory markers.

Plasma samples were thawed overnight at 4°C on ice and centrifuged at 400 *g* for 10 min at 4°C to remove debris. Plasma levels of cytokines IL-18, IL-6, TNF, and of soluble triggering receptor expressed on myeloid cells 2 (sTREM-2) and brain-derived neurotrophic factor (BDNF) were assessed using the Human Neuroinflammatory LEGENDplex Multiplex Assay (BioLegend®) according to the manufacturer's instructions. Briefly, the assay contains allophycocyanin-coated beads conjugated with surface antibodies that allow specific binding to the cytokine of interest. After incubation of the capture beads with the plasma sample, they were stained with biotinylated detection antibodies that bind specifically to the analyte on the capture beads. This creates a capture bead-analyte-detection antibody sandwich which is then stained with streptavidin-phycoerythrin. This final staining provides varying intensity signals based on the amount of bound analyte. Samples were acquired using the Attune NxT Flow Cytometer. The concentration of each individual analyte is determined using a known standard curve using the LEGENDplex™ Data Analysis Online Software Suit. Half of the limit of detection (LOD) was used to perform statistical analyses when a sample value fell below the LOD**.**

**Assessment of fatigue symptoms**

All participants were evaluated using the German version of the Inflammatory Bowel Disease Fatigue (IBD-F) questionnaire, a validated tool for assessing fatigue in UC patients(6).

The IBD-F questionnaire comprises three sections. Part I evaluates fatigue severity and duration (5 items), while Part II assesses the impact of fatigue on daily life (30 items); only these two parts are scored. Part III explores potential contributing factors to fatigue and is intended to support clinical discussion but it is not scored. Both parts I and II use a 0-4 Likert scale. Part I yields a total score ranging from 0-20; a score of 0 indicates no relevant fatigue, in which case the remaining sections are not completed. Part II scores range from 0-120, with adjustments made if “not applicable” responses are selected for specific questions. Fatigue severity was classified as follows: none (0 points in both sections), moderate (1–10 points in Part I or 1–60 points in Part II), and severe (11–20 points in Part I or 61–120 points in Part II). While the IBD-F does not provide validated clinical cutoffs, these thresholds were selected based on the score range and questionnaire structure, as described in the German validation study(6). This stratification enabled exploratory analysis of fatigue in correlation with immune cell profiles, inflammatory markers, and demographic data.

**Statistical analysis**

All data analyses and data visualization were performed using GraphPad Prism 9 and BioRender. Individual values were first tested for normality via Shapiro-Wilk test, after that, parametric data were analyzed using a one-factorial analysis of variance (ANOVA) followed by Tukey’s comparison test. Non-parametric data were analyzed using a Kruskal-Wallis test with Dunn’s multiple comparisons test. Tests were performed between the three studied cohorts: UC patients in active stage, UC patients in remission, and healthy controls. Potential confounders including age, sex and BMI were assessed to ensure they did not significantly differ between groups or influence comparisons. No significant differences were found regarding these variables within groups or during group comparisons. No a priori power calculation was performed; sample size was determined by recruitment availability over the study period. Sex-stratified non-parametric Spearman rank correlations were performed to assess associations between immune cell counts, soluble plasma markers, and IBD-F questionnaire scores (Parts I and II) within each disease group. A Benjamini-Hochberg sensitivity analysis was applied to the correlation p-values within each subgroup matrix; given the small subgroup sizes inherent to sex-stratified analyses, few associations survived correction, consistent with reduced statistical power in exploratory cohorts. Uncorrected p-values are therefore presented, and analyses should be interpreted as hypothesis-generating. Statistical significance was set at *p* < 0.05. Thus, *p* values ≤ 0.05 were reported as follows: * for *p* ≤ 0.05; ** for *p* ≤ 0.01; *** for *p* ≤ 0.001. All data are presented as arithmetic means ± standard deviation (SD).

**Supplementary References**

1. Maaser C, Sturm A, Vavricka SR, et al. ECCO-ESGAR Guideline for Diagnostic Assessment in IBD Part 1: Initial diagnosis, monitoring of known IBD, detection of complications. *J Crohns Colitis*. 2019;13(2):144-164K. doi:10.1093/ecco-jcc/jjy113

2. Dhanda AD, Creed TJ, Greenwood R, Sands BE, Probert CS. Can endoscopy be avoided in the assessment of ulcerative colitis in clinical trials? *Inflamm Bowel Dis*. 2012;18(11):2056-2062. doi:10.1002/ibd.22879

3. Lewis JD, Chuai S, Nessel L, Lichtenstein GR, Aberra FN, Ellenberg JH. Use of the noninvasive components of the mayo score to assess clinical response in Ulcerative Colitis. *Inflamm Bowel Dis*. 2008;14(12):1660-1666. doi:10.1002/ibd.20520

4. Garza AP, Morton L, Pállinger É, et al. Initial and ongoing tobacco smoking elicits vascular damage and distinct inflammatory response linked to neurodegeneration. *Brain Behav Immun Health*. 2023;28:100597. doi:10.1016/j.bbih.2023.100597

5. Gutiérrez-Bautista JF, Rodriguez-Nicolas A, Rosales-Castillo A, et al. Negative Clinical Evolution in COVID-19 Patients Is Frequently Accompanied With an Increased Proportion of Undifferentiated Th Cells and a Strong Underrepresentation of the Th1 Subset. *Front Immunol*. 2020;11. doi:10.3389/fimmu.2020.596553

6. Lehmann JS, Zhao A, Sun B, Jiang W, Ji S. Multiplex Cytokine Profiling of Stimulated Mouse Splenocytes Using a Cytometric Bead-based Immunoassay Platform. *Journal of Visualized Experiments*. 2017;(129). doi:10.3791/56440

7. Scholz KAM, Thomann AK, Teich N, et al. Validation of the German Inflammatory Bowel Disease Fatigue (IBD-F) Questionnaire. *Z Gastroenterol*. 2023;61(2):164-171. doi:10.1055/a-1797-2688
